# Effectiveness of 2D magnesium phosphate hydrogel for surgical decontamination of dental implants: A case series

**DOI:** 10.1111/jopr.13961

**Published:** 2024-10-03

**Authors:** Sara Behmanesh, Kenneth Chow, Jay Dondani, Ashwaq Al‐Hashedi, Faleh Tamimi

**Affiliations:** ^1^ Centre de Spécialistes Dentaires Zeeba Greenfield‐Park Quebec Canada; ^2^ Villa Cathay Care Home Vancouver British Columbia Canada; ^3^ INViCARE Inc Montreal Quebec Canada; ^4^ College of Dental Medicine, Qatar University Doha Qatar

**Keywords:** biofilm management, dental implant, dental implant decontamination, magnesium phosphate, peri‐implantitis

## Abstract

Dental implants, recognized for their enhanced functionality and aesthetic outcomes, are susceptible to peri‐implant mucositis and subsequent peri‐implantitis when oral hygiene is inadequate. Effective biofilm management is critical to prevent and manage these prevalent conditions and promote implant longevity. Materials with a two‐dimensional (2D) structure have demonstrated robust antimicrobial properties. Among these, 2D magnesium phosphates have garnered significant attention due to their additional biocompatibility and osteoconductive properties. This case series explores the application of a thixotropic inorganic hydrogel, composed of 2D magnesium phosphate, in the surgical treatment of dental implant infections. The hydrogel was used for surgical dental implant decontamination in patients diagnosed with peri‐implantitis characterized by inflammation in the peri‐implant mucosa and subsequent progressive loss of supporting bone. The study encompassed eight cases with a history of peri‐implantitis. Clinical measurements were recorded before and after treatment, including bleeding on probing, suppuration, and probing depth. Radiographic evaluations were conducted to assess the exposure of implant threads. The findings revealed a statistically significant decrease in probing depth, bleeding on probing, and the number of exposed implant threads following treatment with the magnesium phosphate hydrogel, though the exact role of the hydrogel in these improvements warrants further exploration.

Dental implants are widely accepted for tooth replacement due to enhanced functionality and aesthetics. However, inadequate oral hygiene can lead to peri‐implant mucositis, an inflammation affecting soft tissues around the implant, potentially progressing to peri‐implantitis characterized by inflammation of supporting hard tissues.^1^ Peri‐implantitis sites exhibit clinical signs of inflammation, bleeding on probing, and/or suppuration, increased probing depths and/or recession of the mucosal margin in addition to radiographic bone loss.^2^

Recent epidemiological studies highlight high prevalence rates of peri‐implant diseases, with over 50% affected by mucositis and 28% to 56% by peri‐implantitis. Bacterial biofilms play a crucial role in disease progression, emphasizing the need for effective implant surface decontamination in prevention and management.^3^ Various methods for decontamination, such as mechanical debridement, antibiotics, and chemical agents, have limitations in eliminating firmly adhered contaminants on titanium surfaces and may contribute to antibiotic resistance concerns.^4^, ^5^, ^6^

In infection control, two‐dimensional (2D) materials have gained attention for their unique structural properties, offering potent antimicrobial effects and suitability for cleaning applications due to extreme thixotropy in gel formation.^7^ Among these materials, magnesium phosphate shows promise in biomedical applications, demonstrating biocompatibility, effective biofilm removal from titanium surfaces in vitro, and bioresorbability with potential for bone regeneration stimulation in vivo.^8^, ^9^

This case series explores the potential benefits of 2D magnesium phosphate in preserving and enhancing peri‐implant health, presenting a novel approach to addressing these challenging clinical conditions.

## Case presentation

Clearance from the Institutional Ethical Committee of the University of Montreal (CERC‐19‐084‐P) was obtained to assess the clinical efficacy of 2D magnesium phosphate in the management of peri‐implant diseases. Each patient provided informed consent. The inclusion criteria included patients with stable dental implants presenting signs of peri‐implantitis1 as defined by the 2017 World Workshop on the Classification of Periodontal and Peri‐Implant Diseases and Conditions.^10^ Exclusion criteria comprised uncontrolled diabetes mellitus, systemic conditions affecting immune function including autoimmune diseases and disorders of bone metabolism, parafunctional oral habits, history of radiation therapy to the head and neck region, use of medications affecting bone metabolism, and tobacco consumption including oral tobacco chewing and smoking.

The study cohort consisted of eight patients recruited from two dental clinics in Montreal (Canada) and Vancouver (Canada), respectively, between September 2022 and July 2023. The sample included^3^ males and^5^ females, with ages ranging from 42 to 68 years old (mean age: 57.5 years) (Table 1). Upon enrollment, each participant underwent an oral examination and peri‐implant health assessment. This evaluation included assessment of suppuration, bleeding on probing, probing depth at six sites per implant (mesiobuccal/mesiolabial, buccal/labial, distobuccal/distolabial, distolingual/distopalatal, lingual/palatal, mesiolingual/mesiopalatal), and extent of implant exposure (quantified by the number of exposed implant threads visible on periapical radiographs). Additional parameters evaluated included implant abutment mobility, occlusal scheme, and prosthesis integrity (including mobility due to abutment screw loosening or loss of luting cement).

**TABLE 1:** Patient demographics, implant region, implant type, surface treatment, and implant manufacturer.

Patient	Age/Sex	Implant region	Implant type	Surface treatment	Implant manufacturer
1	67/F	46	Endosseous	Anodized implant surface	Nobel Biocare
2	62/F	46	Endosseous	Anodized implant surface	Nobel Biocare
3	68/F	15	Endosseous	Anodized implant surface	Nobel Biocare
4	56/F	43	Endosseous	Laser‐Lok microchannels	Biohorizon
5	56/M	33	Endosseous	Laser‐Lok microchannels	Biohorizon
6	45/F	35	Endosseous	Laser‐Lok microchannels	Biohorizon
7	42/M	45	Endosseous	Laser‐Lok microchannels	Biohorizon
8	64/M	14	Endosseous	SLA surface	Straumann

Following the examination, patients received oral prophylaxis of the natural dentition using a piezoelectric ultrasonic scaler. The implant surfaces were debrided using a plastic scaler (Nobelpharma, Nobel Industries, Bethesda, MD) and rinsed with normal saline using a sterile syringe. The eight cases of peri‐implantitis were subsequently treated surgically using 2D magnesium phosphate hydrogel (2D magnesium phosphate [3.02% w/w], sodium phosphate [8.23% w/w], zinc [0.1% w/w]), employing three distinct protocols (Table 2).

**TABLE 2:** Grafting material, decontaminating agent, and disinfection technique in surgical cases.

Protocol	Bone graft material/ technique	Decontaminating agent	Technique
Protocol 1 (Cases 1,2,3)	Allogenic graft (Raptos Allogenic Cortico‐Cancellous blend 0.25 cc, Citagenix Inc., Quebec, Canada).	Saline	After mechanical debridement of the abutment and prosthesis, local anesthesia (Lignocaine with 1:200000 adrenaline) was administered through infiltration using a 27 gauge sterile needle. Full‐thickness peri‐implant mucosal flap was raised using a periosteal elevator. Mechanical debridement of the peri‐implant bone was carried out and sterile gauze to remove any granulation tissue or periosteal tissue remnants. Approximately 1 mL of 2D magnesium phosphate hydrogel (INViCARE Inc., Montreal, Canada) was gradually discharged onto the exposed implant surface using a sterile syringe. After an exposure period of 20 s, the implant surface was mechanically debrided with a sterile rotary nylon brush maintained at 2500 rpm and rinsed with saline. Allogenic graft was then placed onto the site and the flap was approximated and closed with a resorbable suture (4‐0 Vicryl Suture, Ethicon, New Jersey, USA) using single interrupted suturing technique.
Protocol 2 (Cases 4,5,6,7)	Connective Tissue Graft from the palate	Saline + Tetracycline solution	After mechanical debridement of the abutment and prosthesis, the exposed implant threads were treated with tetracycline solution and rinsed with saline. Approximately 1 mL of 2D magnesium phosphate hydrogel (INViCARE Inc., Montreal, Canada) was gradually discharged onto the exposed implant surface using a sterile syringe. After an exposure period of 20 s, the implant surface was mechanically debrided with a sterile rotary nylon brush maintained at 2500 rpm and rinsed with saline. Local anesthesia (Lignocaine with 1:200000 adrenaline) was administered through infiltration using a 29 gauge sterile needle. Connective Tissue Graft harvest was done using the trap door technique from the palate with a coronally positioned flap. Suturing was done with 5‐0 chromic gut sutures and 3/8 16 mm PolyTetraFluoroEthylene sutures (4‐0 PTFE Suture, Ethicon, New Jersey, USA) using single interrupted suturing technique.
Protocol 3 (Case 8)	Enamel matrix derivative (Emdogain, Straumann, Basel, Switzerland) + Cortico‐cancellous allograft + Interpositional vascularized graft	Saline + Tetracycline solution + EDTA	After mechanical debridement of the abutment and prosthesis, local anesthesia (Lignocaine with 1:200000 adrenaline) was administered through infiltration using a 27 gauge sterile needle. Full‐thickness peri‐implant mucosal flap was raised using a periosteal elevator. Mechanical debridement of the peri‐implant bone was carried out with saline soaked sterile gauze to remove any granulation tissue. Exposed implant threads were treated with tetracycline, rinsed with saline then treated with 1 mL of 2D magnesium phosphate hydrogel gel (INViCARE Inc., Montreal, Canada), mechanically debrided with a sterile rotary nylon brush maintained at 2500 rpm and rinsed with saline. Following that, EDTA was applied to the implant threads and subsequently rinsed. Subsequently, the enamel matrix derivative was administered to the defect, followed by the insertion of 1cc of cortico‐cancellous allograft to fill the affected area. The defect was covered with a resorbable collagen membrane (20 × 30 Neomem, Citagenix Inc., Quebec, Canada), a vascularized interpositional periosteal connective tissue graft was placed on top of collagen membrane and primary closure achieved using single interrupted sutures (4‐0 PTFE Suture, Ethicon, New Jersey, USA).

**FIGURE 1:**
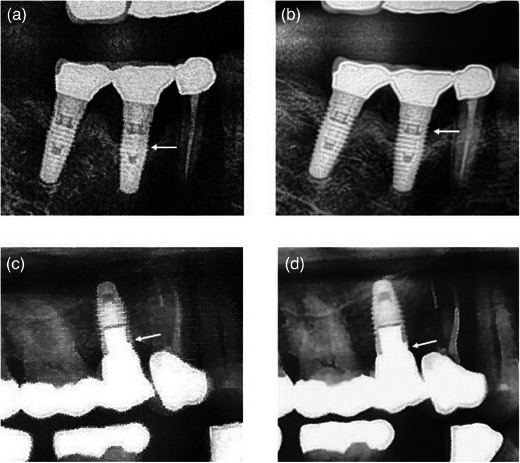
Periapical radiographs of implants at baseline (a), (c) and at follow up after treatment with hydrogel (b), (d). (a) Pretreatment bone attachment level of implant in the region of #46 with exposure of five implant threads. (b) Posttreatment radiographic bone level of implant in the region of #46 with exposure of three implant threads. (c) Pretreatment bone attachment level of implant in the region of #15 with exposure of three implant threads. (d) Posttreatment radiographic bone level of implant in the region of #15 with no exposure of implant threads.

The occurrence of any local adverse effects (irritation, local atopic allergic reactions, taste alterations, numbness, etc.) or systemic reactions (anaphylaxis) was evaluated postoperatively and documented. Postoperative instructions were provided to all patients, and a follow‐up examination was conducted 7 days post surgery to evaluate the surgical site. Bleeding on probing, peri‐implant suppuration, and probing depth were reassessed at the 4‐month follow‐up appointment. Additionally, the number of exposed implant threads visible on intraoral periapical radiographs was measured at the 4‐month follow‐up and compared with baseline values (Figure 1).

## Statistical analysis

The normality of the distribution of baseline and posttreatment data were assessed using the Shapiro–Wilk test. Statistical analysis of data was conducted using SPSS statistical software (Version 29.0.2.0 [20]). Statistically significant differences were set at a *p*‐value of less than 0.05. Mean probing depths and number of sides with positive bleeding on probing and suppuration were calculated for each implant at baseline and posttreatment follow‐up (mean of measurement from six sides) (Table 3). These paired sets of values were compared using Wilcoxon signed‐rank test.

**TABLE 3:** Mean number of sites with bleeding on probing, mean number of sites with suppuration, mean probing depth, and number of implant threads exposed of cases at baseline and posttreatment follow‐up with *p*‐values, respectively.

	*N* of sites with bleeding on probing		*N* sites with suppuration		Mean probing depth (mm)		Number of implant threads exposed on radiograph	
Case no.	Baseline	Posttreatment	Baseline	Posttreatment	Baseline	Posttreatment	Baseline	Posttreatment
01	6	0	0	0	8.0	<1	5	3
02	6	0	0	0	5.5	3.2	2	0
03	6	0	0	0	5.7	3.5	3	0
04	1	0	0	0	3.0	2.7	4	0
05	5	0	0	0	3.7	3.0	4	0
06	1	0	0	0	3.0	2.7	5	0
07	1	0	0	0	3.0	2.7	7	0
08	6	0	6	0	7.7	3.8	3	0
	*p*‐value 0.010		*p*‐value 0.317		*p*‐value 0.011		*p*‐value 0.011	

**FIGURE 2:**
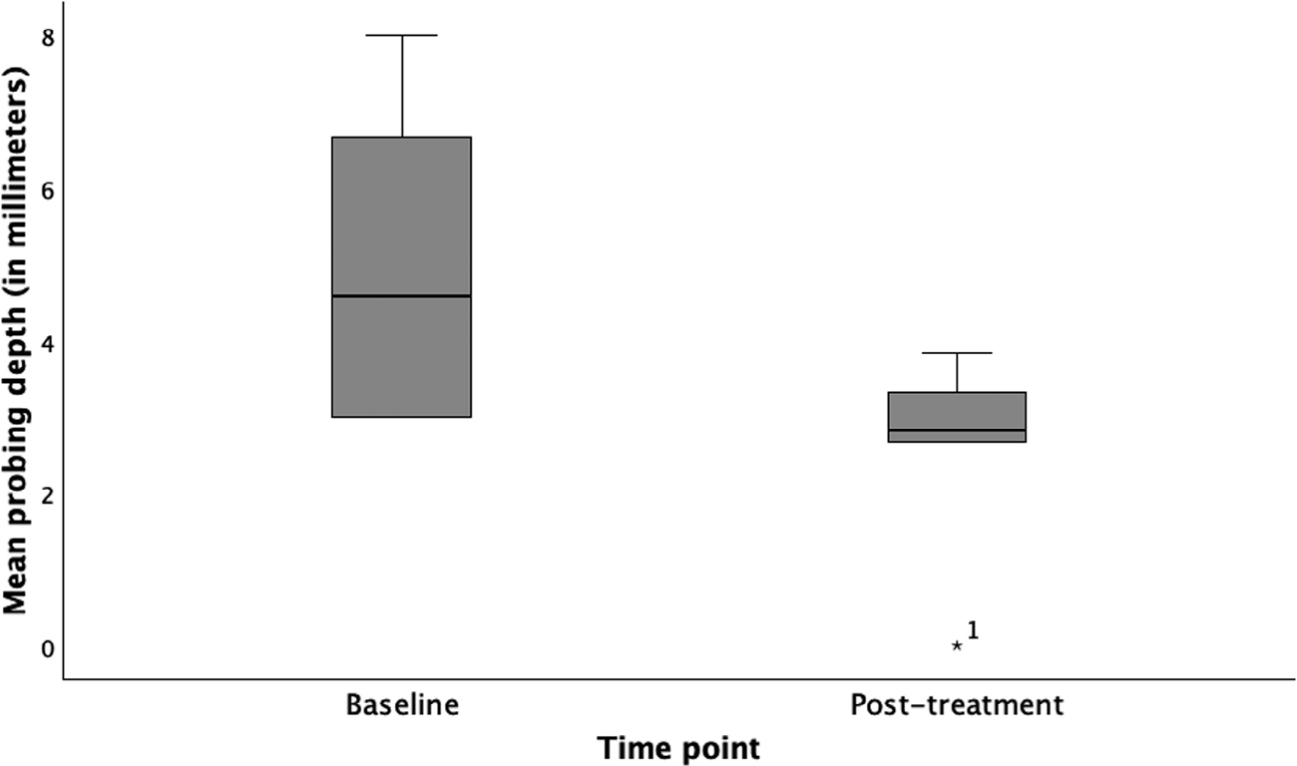
Comparison of mean probing depth of surgical cases (in mm) at baseline and posttreatment follow‐up (*p* = 0.011).

**FIGURE 3:**
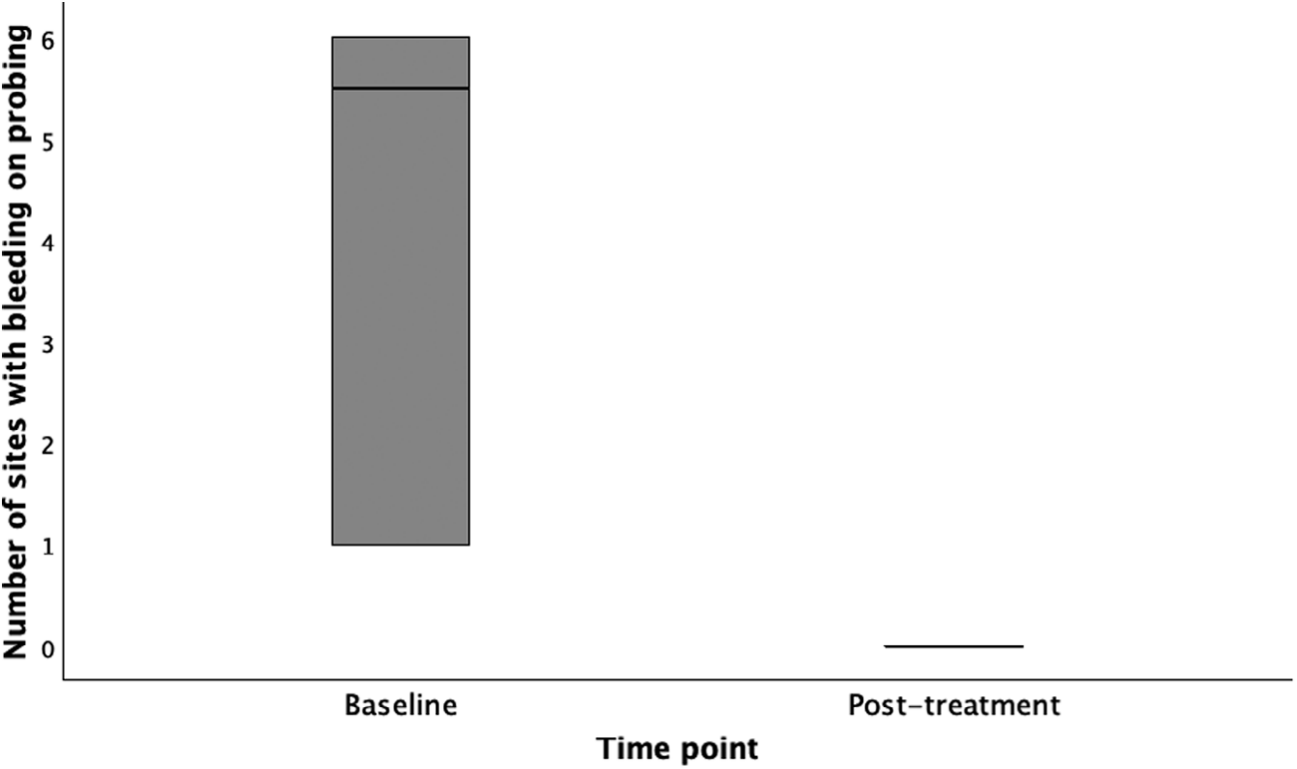
Comparison of number of sites with bleeding on probing of surgical cases at baseline and at posttreatment follow‐up (*p* = 0.010).

**FIGURE 4:**
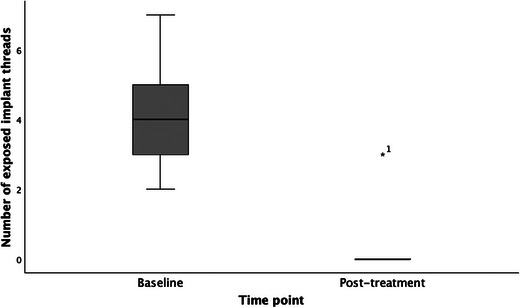
Comparison of number of exposed implant threads of surgical cases at baseline and at posttreatment follow‐up (*p* = 0.011).

There was a statistically significant difference (*p* = 0.011) between the mean probing depths at baseline (4.94 ± 2.09) and posttreatment follow‐up (2.69 ± 1.166), respectively (Figure 2). Additionally, there was a statistically significant difference (*p* = 0.010) between the number of sites with bleeding on probing at baseline (4.00 ± 2.507) and posttreatment follow‐up (0.00 ± 0.00), respectively (Figure 3). There was no statistically significant difference (*p* = 0.317) between the number of sites with suppuration at baseline (0.75 ± 2.121) and posttreatment follow‐up (0.00 ± 0.00). There was a statistically significant difference (*p* = 0.011) between the number of implant threads exposed at baseline (4.13 ± 1.553) and posttreatment follow‐up (0.38 ± 1.061), respectively (Figure 4).

## DISCUSSION

The application of a hydrogel composed of 2D magnesium phosphate nanocrystals for implant decontamination demonstrated promising outcomes in enhancing peri‐implant tissue health. Thfindings revealed improvements in bleeding on probing, probing depths, and radiographic bone level. This case series study showed statistically significant differences in mean probing depths and the number of sites with bleeding on probing between baseline and posttreatment follow‐up. Furthermore, the cases exhibited a significant reduction in exposed implant threads at posttreatment follow‐up compared to baseline as seen radiographically. No adverse effects were observed in association with the hydrogel's use.

The observed reduction in bleeding on probing may be attributed to the cleaning properties of these hydrogels, aligning with previous in vitro research demonstrating that 2D effectively removes biofilms from titanium surfaces without compromising surface topography.^8^ However, the difference in suppuration sites between baseline and posttreatment follow‐up was not statistically significant, which can be attributed to the absence of suppuration at baseline.

The inorganic nanosheets in the composition of hydrogel have previously demonstrated efficacy in decontaminating titanium dental implants.^8^ The optimized magnesium phosphate hydrogel has an alkaline pH of 9.6 and enhances the corrosion resistance of titanium surfaces.^11^ Moreover, its biocompatibility with bone cells and tissues^9^ reduces the risk of implant surface contamination.

In vivo and in vitro studies have shown that magnesium phosphate materials are highly biocompatible with bone tissues and possess potent regenerative properties.^12^ 2D magnesium phosphate has been shown to accelerate bone healing in rats by stimulating osteoblast and osteoclast activity and upregulating runx2 and collagen production.^9^

The reduction in probing depths and absence of bleeding on probing posttreatment indicate improved peri‐implant health and decreased inflammation. These outcomes suggest that magnesium phosphate hydrogel may be a promising adjunct in maintaining oral health for patients with implant‐supported prostheses. The role of magnesium phosphate alone in a surgical setting will have to be evaluated in future studies.

Various techniques have been evaluated for the management of peri‐implantitis, encompassing both nonsurgical and surgical approaches.^13^ In surgical approaches, the role of implant decontamination was found to be more important than the bone regenerative material used in the procedure for radiographic bone level gain. This importance was more pronounced in implants with rough micro geometries compared to smooth implants.^14^, ^15^ The adjunctive disinfectants used in the surgical protocol, EDTA, Tetracycline, and Saline have been shown to effectively decontaminate the implant surface, with EDTA and saline treatment also showing an increase in re‐osseointegration.^13^, ^16^, ^17^, ^18^, ^19^ Interestingly, a more profound decrease in the number of sites with bleeding on probing and probing depths was achieved with Protocol 1, where only saline and magnesium phosphate gel were used with no adjunctive disinfectants or antibiotics. A conclusive proof of the relative effectiveness of these agents will require a posthoc analysis of a study with a larger sample size. The grafts used in Protocols 1 and 3, Emdogain and cortico‐cancellous allograft, have been proven to increase bone attachment,^20^, ^21^ although some studies have indicated that the utilization of bone filler material did not yield significant enhancements in terms of re‐osseointegration and bone level augmentation.^15^ Therefore the utility of these grafts should be further evaluated. The improvement in radiographic bone level could be related to the osteoinductive properties of the hydrogel, although this hypothesis would have to be further explored in future studies.

This study has several limitations. Peri‐implantitis cases in the present case series were treated surgically with three different mechanical/chemical de‐contamination protocols, bone substitutes, or connective tissue grafts. Two of these protocols included the use of antibiotics, and all of them included the use of additional antiseptic agents. In addition, two protocols also involved the use of bone graft materials. The difference in bone grafts used between the surgical cases may act as a confounding factor in the increase of bone attachment levels. Therefore, it is not possible to infer the current study's findings on the effect of the magnesium phosphate alone (in other words, the same surgical interventions without magnesium phosphate would also result in peri‐implant disease remission) and therefore cannot confirm the role of magnesium phosphate per se on bleeding on probing or bone regeneration. Based on the present case series design and data, it could be suggested that magnesium sulfate seemed not to interfere with surgical site healing, but its effect on peri‐implant disease resolution is not clear. In addition, the dentists involved in the study were not blinded and were not calibrated. Future RCTs with appropriate control groups would be needed to assess this.

In conclusion, this case series reveals no negative effects associated with the use of the magnesium phosphate hydrogel and provides valuable information for future randomized clinical trials. The results observed in this study warrant further investigation into the potential of 2D magnesium phosphate hydrogels as a novel approach for managing peri‐implant diseases.

## CONCLUSION

The application of 2D magnesium phosphate hydrogel in this case series was associated with improvements in peri‐implant tissue health, including reductions in probing depths and bleeding on probing at the 4‐month follow‐up. A statistically significant reduction in exposed implant threads with radiographic bone fill was observed, though the specific contribution of magnesium phosphate to bone regeneration requires further exploration. Nonetheless, the hydrogel did not impede surgical site healing, and further studies are warranted to explore its potential role in managing peri‐implant diseases (SUPPORTING INFORMATION).

## CONFLICT OF INTEREST STATEMENT

Dr. Jay Dondani serves as the Clinical Technology Specialist at INViCARE Inc. Dr. Ashwaq Al‐Hashedi is the Founder and Chief Executive Officer at INViCARE Inc. Dr. Faleh Tamimi is the Chief Scientific Officer at INViCARE Inc. Dr. Hashedi and Dr. Tamimi hold equity in INViCARE Inc. The 2D magnesium phosphate hydrogel used in the case series is a proprietary product of INViCARE Inc.

## Supporting information



Supporting Information
