# Supplementary material for: Effectiveness of 2D magnesium phosphate hydrogel for surgical decontamination of dental implants: A case series
Source: J Prosthodont. 2024 Oct 3;34(5):449–55. doi: 10.1111/jopr.13961 (PMC12147417; doi:10.1111/jopr.13961)
Supplement: Supplementary file 1 — Supporting Information [file JOPR-34-449-s001.docx]

# RAW DATA

Baseline and post-treatment bleeding on probing, suppuration, probing depth and number of threads exposed on radiograph respectively of surgical cases.

| **Patient** | **A/S** | **Implant region** | **Implant** | **Aspect** | **Probing depth** | | **Bleeding on probing** | | **Suppuration** | | **Number of threads exposed on radiograph (Highest value as visible on mesial or distal aspect)** | |
| --- | --- | --- | --- | --- | --- | --- | --- | --- | --- | --- | --- | --- |
| 1 | 67/F | 46 | Nobel Biocare | ML/MP | 7 | <1 | P | A | A | A | 5 | 3 |
|  |  |  |  | MB | 9 | <1 | P | A | A | A |  |  |
|  |  |  |  | B | 9 | <1 | P | A | A | A |  |  |
|  |  |  |  | DB | 8 | <1 | P | A | A | A |  |  |
|  |  |  |  | DL/DP | 9 | <1 | P | A | A | A |  |  |
|  |  |  |  | L/P | 6 | <1 | P | A | A | A |  |  |
| 2 | 62/F | 46 | Nobel Biocare | ML/MP | 4 | 2 | P | A | A | A | 2 | 0 |
|  |  |  |  | MB | 8 | 4 | P | A | A | A |  |  |
|  |  |  |  | B | 8 | 5 | P | A | A | A |  |  |
|  |  |  |  | DB | 4 | 2 | P | A | A | A |  |  |
|  |  |  |  | DL/DP | 3 | 3 | P | A | A | A |  |  |
|  |  |  |  | L/P | 6 | 3 | P | A | A | A |  |  |
| 3 | 68/F | 15 | Nobel Biocare | ML/MP | 3 | 3 | P | A | A | A | 3 | 0 |
|  |  |  |  | MB | 7 | 3 | P | A | A | A |  |  |
|  |  |  |  | B | 6 | 3 | P | A | A | A |  |  |
|  |  |  |  | DB | 6 | 4 | P | A | A | A |  |  |
|  |  |  |  | DL/DP | 6 | 4 | P | A | A | A |  |  |
|  |  |  |  | L/P | 6 | 4 | P | A | A | A |  |  |
| 4 | 56/F | 43 | Biohorizon | ML/MP | 3 | 3 | A | A | A | A | 4 | 0 |
|  |  |  |  | MB | 3 | 2 | A | A | A | A |  |  |
|  |  |  |  | B | 4 | 3 | P | A | A | A |  |  |
|  |  |  |  | DB | 3 | 3 | A | A | A | A |  |  |
|  |  |  |  | DL/DP | 2 | 2 | A | A | A | A |  |  |
|  |  |  |  | L/P | 3 | 3 | A | A | A | A |  |  |
| 5 | 56/M | 33 | Biohorizon | ML/MP | 4 | 3 | P | A | A | A | 4 | 0 |
|  |  |  |  | MB | 5 | 3 | P | A | A | A |  |  |
|  |  |  |  | B | 4 | 3 | P | A | A | A |  |  |
|  |  |  |  | DB | 3 | 3 | P | A | A | A |  |  |
|  |  |  |  | DL/DP | 3 | 3 | A | A | A | A |  |  |
|  |  |  |  | L/P | 3 | 3 | P | A | A | A |  |  |
| 6 | 45/F | 35 | Biohorizon | ML/MP | 3 | 3 | A | A | A | A | 5 | 0 |
|  |  |  |  | MB | 3 | 2 | A | A | A | A |  |  |
|  |  |  |  | B | 4 | 3 | P | A | A | A |  |  |
|  |  |  |  | DB | 3 | 3 | A | A | A | A |  |  |
|  |  |  |  | DL/DP | 2 | 2 | A | A | A | A |  |  |
|  |  |  |  | L/P | 3 | 3 | A | A | A | A |  |  |
| 7 | 42/M | 45 | Biohorizon | ML/MP | 3 | 3 | A | A | A | A | 7 | 0 |
|  |  |  |  | MB | 3 | 2 | A | A | A | A |  |  |
|  |  |  |  | B | 4 | 3 | P | A | A | A |  |  |
|  |  |  |  | DB | 3 | 3 | A | A | A | A |  |  |
|  |  |  |  | DL/DP | 2 | 2 | A | A | A | A |  |  |
|  |  |  |  | L/P | 3 | 3 | A | A | A | A |  |  |
| 8 | 64/M | 14 | Straumann | ML/MP | 6 | 4 | P | A | P | A | 3 | 0 |
|  |  |  |  | MB | 7 | 3 | P | A | P | A |  |  |
|  |  |  |  | B | 10 | 4 | P | A | P | A |  |  |
|  |  |  |  | DB | 10 | 4 | P | A | P | A |  |  |
|  |  |  |  | DL/DP | 7 | 4 | P | A | P | A |  |  |
|  |  |  |  | L/P | 6 | 4 | P | A | P | A |  |  |
|  |  |  |  | ML/MP |  |  |  |  |  |  |  |  |
